# Comparison of the modified fluorescent method and conventional Ziehl–Neelsen method in the detection of acidfast bacilli in lymphnode aspirates

**DOI:** 10.4103/1742-6413.53887

**Published:** 2009-07-18

**Authors:** Vamseedhar Annam, Mohan H Kulkarni, Rekha B Puranik

**Affiliations:** Department of Pathology, Sree Siddhartha Medical College & Research Center, Tumkur, India; 1 Department of Pathology, Karnataka Institute of Medical Sciences, Hubli, Karnataka, India

**Keywords:** Cytology, fluorescent method, tuberculosis, Ziehl–Neelsen stain

## Abstract

**Objectives:**

The objectives were to correlate the modified fluorescent method with the conventional Ziehl–Neelsen (ZN) method for the detection of acid-fast bacilli (AFB) and, also to study the efficacy and advantages of using the auramine–rhodamine stain on lymph node aspirates under fluorescent microscopy.

**Methods:**

In 108 consecutive patients with a clinical suspicion of tuberculosis (TB) presenting with lymphadenopathy, fine needle aspirations were performed. Smears from the aspirates were processed for routine cytology, the conventional ZN method, and the modified fluorescent method. The significance of the modified fluorescent method over the conventional ZN method was analyzed using the chi-square test.

**Results:**

Out of 108 aspirates, 102 were studied and remaining 6 were excluded from the study due to diagnosis of malignancy in 4.04% (4/6) and inadequate aspiration in 2.02% (2/6). Among the 102 aspirates, 44.11% (45/102) were positive for AFB on the conventional ZN method, 58.9% (60/102) were indicative of TB on cytology, while the smear positive increased to 81.37% (83/102) on the modified fluorescent method.

**Conclusions:**

Fluorescent microscopy has the advantage of speed and ease of screening, and reduces observer fatigue. The modified fluorescent method was found to be more advantageous than routine cytology and conventional ZN method, particularly in paucibacillary cases. The bacillary positivity rates were higher in the modified fluorescent method than in the ZN method. Hence, the modified fluorescent method can be an adjuvant when used with routine cytology for the identification of AFB.

## INTRODUCTION

Tuberculosis (TB) continues to be a major health problem in developing countries. Lymphadenopathy is the most common presentation of extrapulmonary TB.[1,2] The clinical parameters for the diagnosis of TB in lymph nodes are neither specific nor do their absence exclude TB involvement.[3,4] Fine-needle aspiration cytology (FNAC) of lymph nodes in TB has varied cytomorphological features. However, the conventional Ziehl–Neelsen (ZN) method for acid-fast bacilli (AFB) plays a key role in the diagnosis and also for the monitoring of treatment in TB. Its major disadvantage is low sensitivity ranging from 20% to 43%.[5,6] Mycobacterial culture is the reference method for the detection of tubercle bacilli but it is time consuming and requires specialized safety procedures in laboratories. Serological techniques have the disadvantage of lack of sensitivity and specificity.[5] Newer molecular techniques such as polymerase chain reaction (PCR), although rapid, are costly to be routinely used in developing countries where most TB cases occur.[7] Hence, a method for the identification of AFB which is more sensitive than the ZN method is required for early detection of TB. Also there is no previous literature related to the comparison of Auramine–Rhodamine (AR)-stained smears under fluorescent microscopy with ZN stain for the detection of AFB in lymph node aspirates. Thus, the present study was undertaken with the following objectives:

1. to correlate the modified fluorescent method with the conventional ZN method, and to also compare the results with routine cytology on lymph node aspirates for the detection of AFB;
2. to study the efficacy and advantages of using AR stain under fluorescent microscopy.

## MATERIALS AND METHODS

One hundred and eight consecutive patients suspected clinically of having TB with lymphadenopathy referred for FNAC to the Department of Cytology from April 2003 to September 2004 were included in the study. Exclusion criteria were recurrent/episodic cough without fever and recurrent fever with an intervening normal period. Relevant investigation details, such as hematology, chest radiogram, were reviewed in these patients. All the aspirates by FNAC were processed for direct microscopy using conventional ZN staining and routine cytology, and compared with the findings of the modified fluorescent method. A total of three smears were prepared from each of the FNAC aspirates: one alcohol-fixed wet smear was stained by hematoxylin and eosin (H and E) for cytological examination directly, and second and third air-dried smears were stained with ZN and AR stains, respectively. When the aspirate was scanty, only one smear was prepared and examined for cytology followed by both AR and ZN techniques.

Depending upon the cytomorphological appearances, tuberculous lymphnodes were subdivided into four categories: (i) purulent with caseation; (ii) only caseation; (iii) caseation with epithelioid cells; and (iv) noncaseating with epithelioid cells.

The following modified fluorescent staining procedure was implemented:

1. The heat-fixed smears were stained with the filtered AR mixture at 37°C for 15 minutes.
2. The slide was rinsed with de$iodinized water for 2 minutes.
3. Decolorization was performed with 0.5% hydrochloric acid in 70% ethanol for 2 minutes.
4. The slide was rinsed with deiodinized water for 2 minutes.
5. Counterstaining was performed with 0.5% aqueous potassium permanganate for 2 minutes.
6. The slide was rinsed with deiodinized water for 2 minutes, and air dried and examined under high power (×400) which was confirmed under oil immersion (×1000).

The AFB appear as yellow to orange, slender, rod-shaped bacilli under fluorescent microscopy. Smears stained by the conventional ZN method, directly, were examined for AFB under oil immersion (×1000) using light microscopy.

The data were processed using test of association (chi-square test).

## RESULTS

A total of 108 fine-needle aspirated specimens from lymph nodes were included in the study. Of these, 102 specimens were evaluated and the remaining 6 were eliminated because four aspirates identified malignancy and two were inadequate. A total of 20 patients were HIV positive. The age ranged from 2 to 70 years, with the mean age of 23.4 ± 13.5 years. Female preponderance was noted accounting for 61.7% (63/102) of cases. Among the 102 lymph nodes studied, aspirates were from cervical (*n* = 76), inguinal (*n* = 11), and axillary (*n* = 15) groups.

Of the 102 aspirates, the smear positivity for AFB on the conventional ZN method was 44.11% (45/102) while the positivity increased to 81.37% (83/102) on the modified fluorescent method. The correlation between the conventional ZN method and the modified fluorescent method [Table 1] showed statistical significance (χ^2^ = 18.63, df = 1, *P*<0.001). The cytomorphological features observed were reactive lymphadenitis in 26.5% (27/102) cases, acute suppurative lymphadenitis in 14.7% (15/102) cases and tubercular lymphadenitis in 58.9% (60/102) cases.

**Table 1:** Comparison of the conventional ZN method with the modified fluorescent method for the detection of acid-fast bacilli

*Conventional ZN method*	*Modified fluorescent method*		*Total*
		
	*Positive*	*Negative*	
Positive	44	01	45
Negative	39	18	57
Total	83	19	102

Statistical significance: χ^2^ = 18.63, df = 1, *P*< 0.001

The criteria for the diagnosis of reactive lymphadenopathy was established based on polymorphic population of lymphoid cells without malignant features and a considerable number of tingible body macrophages.[8] Out of 26.5% (27/102) cases diagnosed as reactive lymphadenitis, the modified fluorescent method was positive for AFB in 40.74% (11/27) cases, and all the cases were negative by the conventional ZN method. Of the 20 HIV-infected patients, a reactive pattern on cytology was seen in 12 cases. Among these 12, the modified fluorescent method was positive for AFB in 9 cases and all the cases were negative by conventional the ZN method.

The cytomorphological diagnosis of acute suppurative lymphadenitis was based on the aspirated purulent material showing abundant neutrophils with macrophages containing ingested necrotic debris in a necrotic background. Among 14.7% (15/102) cases diagnosed as acute suppurative lymphadenitis, the modified fluorescent method was positive in 86.67% (13/15) cases while the conventional ZN method identified AFB in 13.33% (2/15) cases.

On cytomorphology, the tuberculous lymph node, was diagnosed using the following criteria: (i) purulent with caseation; (ii) only caseation; (iii) caseation with epithelioid cells; and (iv) noncaseating with epithelioid cells.[9] Out of 58.9% (60/102) cases, AFB were identified by the modified fluorescent and conventional ZN methods in 98.33% (59/60) and 70% (42/60) cases, respectively. Of the eight HIV-infected patients, a tubercular pattern on cytology was seen in three cases. All the cases were positive by both conventional ZN method and modified fluorescent method.

The comparison of the results of the fluorescent method and the conventional ZN method with routine cytology is emphasized [Table 2].

**Table 2:** Correlation of the cytomorphological diagnosis with the modified fluorescent method and the conventional ZN method

*Cytomorpho-logical diagnosis*	*Modified fluorescent method*		*Conventional ZN method*		*Total*
			
	*Positive*	*Negative*	*Positive*	*Negative*	
Reactive LN	11	16	00	27	27
Suppurative LN	13	02	03	12	15
TB LN	59	01	42	18	60
Total	83	19	45	57	102

## DISCUSSION

India has a long history of research and demonstration projects on TB.[10] The detection of AFB is often considered as the evidence of the infected state. Thus, the laboratory plays a critical role in the diagnosis of TB.[11] In developing countries, microscopy of the specimen is by far the fastest, cheapest, and most reliable method for the detection of AFB.

Since the early 1940s, the comparison of the fluorescent method with the conventional ZN method on sputum smears was implemented to improve the smear positivity for the detection of AFB. The use of a fluorochrome acid-fast stain, such as AR, is recommended because of its increased sensitivity and ease of interpretation compared with the ZN method.[12] The accepted practice is to stain sputum smears with AR at room temperature.[13–15] The present study was our first attempt to use the modified fluorescent method and compare it with the conventional ZN method on lymph node aspirates (in cytology). The staining of these lymph node smears with AR at 37°C for 15 minutes increased the smear positivity by 37.26% over the conventional ZN method. Staining at 37°C is no more difficult than staining at room temperature and requires only that the AR stain be prewarmed prior to use.

**Figure 1:**
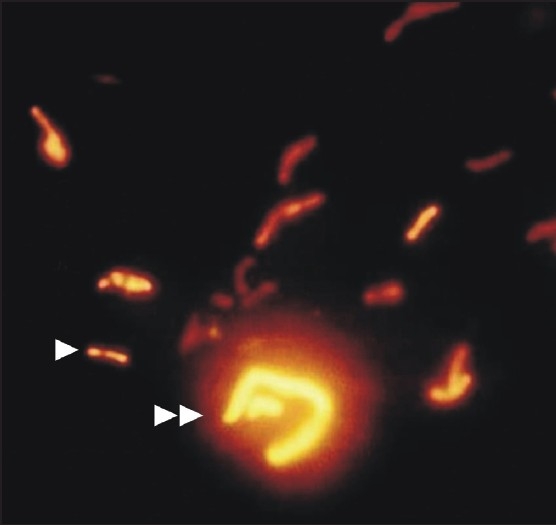
Smear showing acid fast bacilli typically fluorescing as golden, slender, rod-shaped bacilli (single arrow head). Also seen are some naturally fluorescent particles (double arrow head) adjacent to bacilli (AR, ×1000)

**Figure 2:**
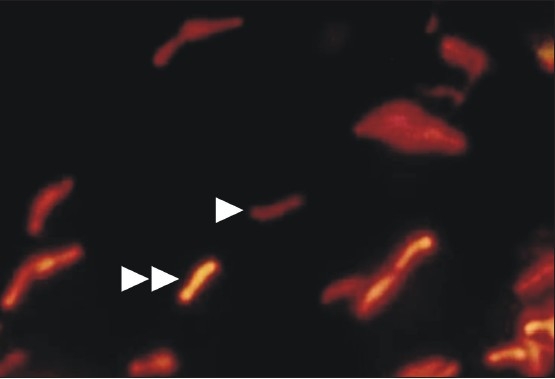
Smear showing viable acid fast bacilli appearing with more intense fluorescence (double arrow head) when compared with some acid fast bacilli (single arrow head) appearing less fluorescence (AR, ×1000)

The AFB typically fluoresce as golden, slender, rod-shaped bacilli, but they may appear curved or bent [Figure 1.] Also, some individual AFB may display heavily stained areas referred to as beads and/or alternating light and dark areas of stain producing a banded appearance. Although the ability to retain aryl methane dyes, such as auramine O, after washing with alcohol or weak acids is a primary feature of the genus Mycobacterium, it is not entirely unique to the genus. Other bacteria, which contain mycolic acids, such as Nocardia, can also exhibit this feature. The exact method by which the stain is retained is unclear but it is thought that the stains become trapped within the cell or may form a complex with the mycolic acids. This is supported by the finding that shorter chain mycolic acids or Mycobacterial cells with disrupted cell walls stain weakly acid-fast. A disadvantage is that there is a more intense binding of the mycolic acids to the fluorochrome dye causing bacilli, which are apparently rendered nonviable by chemotherapy to be acid-fast [Figure 2].[16,17] However, good observation is required to distinguish with certainty AFB from other small, naturally fluorescent particles present in some smears [Figure 1]. When first using fluorescent microscopy, it is necessary to examine all small fluorescent objects seen both with the ×10 and ×40 objectives. With practice, it becomes possible to distinguish bacilli with a fair degree of certainty under the × 10 objective only, so that almost all negative smears can be examined with this objective only. However, it is always necessary to confirm the bacillary morphology with the higher power when the smears contain a low density of bacilli. Finally, if any doubt remains, it is possible to ring individual suspicious objects with the diamond objective marker, then restain with the fluorescence stain by the ZN method and then finally examine with an oil-immersion lens.

Previous studies on sputum smears have shown the positivity rates for both the ZN and AR methods as 65% and 80%, respectively.[14] But, in our study on lymph node aspirates in cytology, the positivity rates were 44.11% for the ZN method and 81.37% for the modified fluorescent method (*P*<0.001). The discrepancies between the cytomorphological diagnosis and the modified fluorescent method in the present study occurred in 25 specimens. Out of 25 specimens, 11 specimens were reactive lymphadenitis, 13 specimens were acute suppurative lymphadenitis but these specimens were positive for AFB by the modified fluorescent method, and 1 specimen was negative for AFB by the modified fluorescent method but diagnosed as TB on cytology. The possible explanation for the diagnosis of specimens as reactive lymphadenitis on cytology but positive for AFB by the modified fluorescent method may be the absence of scattered epithelioid cells among the polymorphous population of lymphoid cells and also the decrease in the density of the bacilli. All these AFB-positive patients responded well to the antitubercular therapy. Among the 13 specimens diagnosed as suppurative lymphadenitis but positive for AFB by the modified fluorescent method, the probable reason could be the absence of the bacilli amidst the necrotic debris. Also, one specimen diagnosed as TB on cytology and negative by the modified fluorescent method may be due to the decrease in the density of the bacilli.

We conclude that the modified fluorescent method is more sensitive than the conventional ZN method. The advantage of using the fluorescent staining method is that fluorescent-stained slides can be examined under low magnification allowing for much larger areas of the smear to be examined in a short period of time. The use of the modified fluorescent method greatly improves the diagnostic value especially in patients with a low density of bacilli that are likely to be missed on ZN-stained smears. Also, the use of AR staining alone could not be an alternative method to conventional ZN staining. Hence, it could be beneficial when the modified fluorescent method is used as an adjuvant along with clinical parameters and cytological features in lymph node aspirates.

## COMPETING INTEREST STATEMENT BY ALL AUTHORS

No competing interest to declare by any of the authors.

## AUTHORSHIP STATEMENT BY ALL AUTHORS

All authors of this article declare that we qualify for authorship as defined by ICMJE http://www.icmje.org/#author.

Each author has participated sufficiently in the work and take public responsibility for appropriate portions of the content of this article.

Each author acknowledges that this final version was read and approved.

## ETHICS STATEMENT BY ALL AUTHORS

This study was conducted with approval from Institutional Review Board (IRB) (or its equivalent) of all the institutions associated with this study. Authors take responsibility to maintain relevant documentation in this respect.
